# Computing Persistent Homology by Spanning Trees and Critical Simplices

**DOI:** 10.34133/research.0230

**Published:** 2023-09-14

**Authors:** Dinghua Shi, Zhifeng Chen, Chuang Ma, Guanrong Chen

**Affiliations:** ^1^ Department of Mathematics, College of Science, Shanghai University, Shanghai, China.; ^2^ School of Big Data, Fuzhou University of International Studies and Trade, Fuzhou, China.; ^3^ Department of Data Science and Big Data Technology, School of Internet, Anhui University, Hefei, China.; ^4^ Department of Electrical Engineering, City University of Hong Kong, Hong Kong, China.

## Abstract

Topological data analysis can extract effective information from higher-dimensional data. Its mathematical basis is persistent homology. The persistent homology can calculate topological features at different spatiotemporal scales of the dataset, that is, establishing the integrated taxonomic relation among points, lines, and simplices. Here, the simplicial network composed of all-order simplices in a simplicial complex is essential. Because the sequence of nested simplicial subnetworks can be regarded as a discrete Morse function from the simplicial network to real values, a method based on the concept of critical simplices can be developed by searching all-order spanning trees. Employing this new method, not only the Morse function values with the theoretical minimum number of critical simplices can be obtained, but also the Betti numbers and composition of all-order cavities in the simplicial network can be calculated quickly. Finally, this method is used to analyze some examples and compared with other methods, showing its effectiveness and feasibility.

## Introduction

In the era of big data [1], higher-dimensional data and their analysis connect together the fields of data science [2,3], network science [4], and computational topology [5,6]. In these studies, algebraic topology especially persistent homology [7–10] plays a key role. In fact, the shape of a dataset can be viewed as the real representation of the true data only if it appears persistently in various spatiotemporal scales; otherwise, it might likely contain sampling errors or noise.

**Fig. 1.**
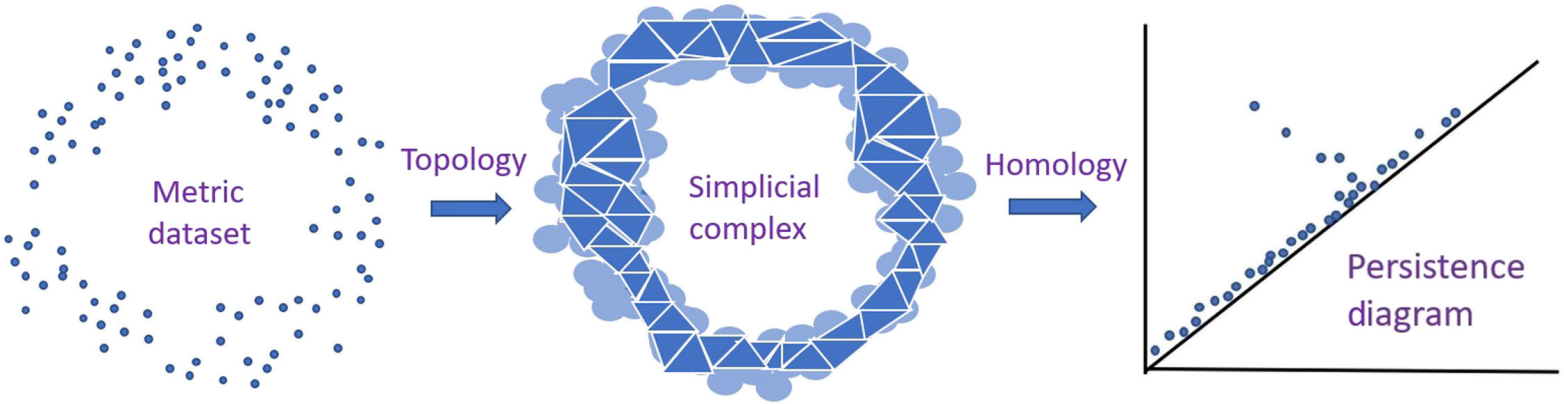
Point-cloud data and their topological features [9].

One typical example is the point-cloud data, such as the recorded spatial coordinates and brightness of a scanned image, as shown in Fig. 1 (left picture). With this dataset, with a given threshold value, one can establish the topological relationship among all the points, obtaining a simplicial complex as shown in Fig. 1 (middle picture). Simple complexes obtained by different methods are usually different, for example, they can be dense such as the Čech complex [11] and the Vietoris-Rips complex [12], or sparse such as the Alpha complex [13] and Witness complex [14]. From the perspective of network science, the network composed of all-order (all different orders of) simplices in a simplicial complex is called a simplicial network. To this end, by increasing the threshold values, one can obtain and analyze the filtered simplicial network so as to dynamically extract its topological features under different scales, as shown in Fig. 1 (right picture). In general, time series data can be converted to point-cloud data after determining the number of points, time windows, and spatial dimensions [15].

**Fig. 2.**
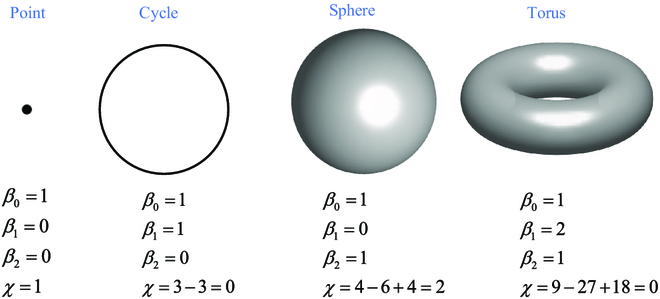
Betti numbers of a node, a cycle, a sphere, and a torus [19] and their characteristic number.

Network science can be traced back to Euler when he solved the Königsberg 7-bridge problem, thereby establishing the graph theory. In the era of the Internet with big data, the models of small-world networks [16] and scale-free networks [17] attracted a lot of attention. Consequently, in the study of totally homogeneous networks [18], it was found that the Euler characteristic number *χ* can be applied to analyzing complex networks, where cliques (fully connected subnetworks) are simplices of different orders, such as node (0-simplex, i.e., order 0), edge (1-simplex), triangle (2-simplex), and so on. The summation of these clique numbers with alternative signs is an invariant value of the given network, namely, the Euler characteristic number *χ*. On the other hand, in addition to cliques, there are many cavities in a large-scale network. The shape and the number of some cavities commonly seen in geometry are shown by the examples in Fig. 2. In order to distinguish the numbers of cavities on a sphere and on a torus, Poincaré introduced the concept of triangulation, to triangulate a cycle to get a triangle that is not a 2-simplex, to triangulate a sphere to get a tetrahedron that is not a 3-simplex, and to triangulate a torus to get a more complex network, which will be further discussed in the Methods section. The numbers of cavities are called Betti numbers, denoted *β_k_*, and the summation of the Betti numbers with alternative signs is equal to *χ* (see Fig. 2).

One conventional approach to network science studies is to consider simplices as basic network elements and define their vector spaces, in which chains, cycles, etc. are further introduced, and consequently apply the theory and methods of algebraic topology. Here, these networks are called simplicial (complexes [19]) networks [20], which are higher-order networks [21,22], and their orders are determined by the highest-order simplex. Thus, the common graph networks are 1-order ones because they only consider nodes and edges. It is known that the cyclic structures in simplicial networks are of fundamental importance because they provide feedback paths in higher-order dynamical interactions over such networks [23]. However, for relatively dense networks, the numbers of cycles are extremely large. Therefore, classifying cycles in large simplicial networks is a great challenge, which eventually relies on the computation of homology groups [24]. In addition to Betti numbers, which can only give the ranks of homology groups, the cycles whose classes constitute the elements of the homology groups carry some important information. In practice, interests are usually in representative cycles that have some optimal properties.

In homology theory, persistent homology starts from the Morse theory [25], which uses a continuous real variable function to calculate homology. Barannikov [26] studied framed Morse complex and its invariants. Forman [27] discredited Morse functions and assigned real values to simplices in simplicial networks, thereby classifying them. It was revealed [26,27] that the number of critical simplices (see the “Basic concepts” section for a precise definition) is between the number of cliques and the number of cavities, and that the summation of the numbers of critical simplices with alternative signs is also equal to the characteristic number *χ*. There are several different ways to assign values to simplices; for example, Sizemore [28] used the edge weights of a weighted network, Horak et al. [29] assigned values to simplices from lower order to higher order, and Kannan et al. [30] designed a method that can theoretically find a near-minimum number of critical simplices.

**Fig. 3.**
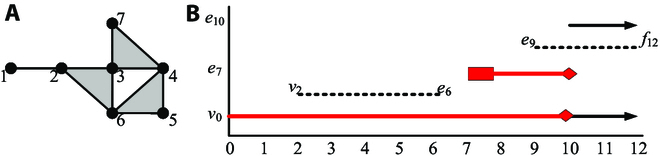
(A) A 2-order simplicial network with 3 shaded triangles. (B) Betti persistence barcodes of the simplicial network with different Morse values.

Similar to the point-cloud data scenario where different thresholds can induce a filtered simplicial network, by assigning values of a discrete Morse function to simplices, one can also obtain a sequence of nested simplicial subnetworks [31,32]. However, different methods for assigning values will result in different filtered networks with different numbers of critical simplices. The results of computing persistent homology can be represented by persistence diagrams, persistence barcodes, persistence landscapes, and so on. However, the persistence barcodes of different Morse values are different too, as can be seen from Fig. 3 in the Methods section.

This paper proposes an optimal method for assigning Morse values by using all-order spanning trees of the network, such that the number of critical simplices in each order is equal to the Betti number of the same order. Furthermore, by solving the systems of spanning trees and critical simplex equations, those simplices that constitute all-order cavities can be easily obtained because the corresponding equations have unique solutions.

The rest of the paper is organized as follows. In the Results section, the new method will be applied to some networks, specifically the *C. elegans* neural network, BA scale-free model network, and a network formed by point-cloud dataset, which will be compared with the existing methods to demonstrate the effectiveness and feasibility of the new method. In the Discussion section, different sequences of nested networks, the optimal property of the obtained cavities, and the influence of various chains on homology calculation will be discussed. Finally, in the Methods section, homology of simplicial network and discrete Morse function will be introduced, where an example is given to show how to use spanning trees to assign Morse function values to simplicial networks, and to draw persistence barcodes. Then, computational methods based on the systems of spanning trees and critical simplex equations will be developed.

## Results

The procedure shown in the Methods section is summarized as follows. Firstly, a simplicial network is obtained by calculating or enumerating all simplices. Secondly, in the simplicial network, a sequence of nested simplicial subnetworks (i.e., filtered simplicial network) is obtained by searching all-order spanning trees and assigning Morse function values to simplices. Finally, in different subnetworks, simplices composed of all-order cavities are obtained by solving matrix equation (*T − **B**_k_*) (*T − **x***) *^T^* **=** (*C − **B**_k_*) (mod 2).

Now, the developed method is applied to the *C. elegans* neural network, BA scale-free network, and Stanford Dragon graphic network. The results will be compared with the existing methods.

### *C. elegans* neural network [33]

As a conventional network *G* = {*V*, *E*}, it has 297 neurons in *V* and 2,148 synapses in *E*, and the adjacency matrix is given by the dataset from Ref. [33].

By computing the conventional network *G* [24], the following simplicial network ***K*** = {*V*, *E, T*, *•••*} can be obtained, with the Euler characteristic number (and the Betti numbers *β_k_*) *χ* **=** 297 – 2,148 + 3,241 – 2,010 + 801 – 240 + 40 − 2 (**=** 1 – 139 + 121 − 4) **=** −21. The ranks of boundary matrices ***B***_1_, ***B***_2_, *•••*, ***B***_7_ for describing the relationship between simplices are *r*_1_ = 296, *r*_2_ = 1,713, *r*_3_ = 1,407, *r*_4_ = 599, *r*_5_ = 202, *r*_6_ = 38, and *r*_7_ = 2, respectively. Then, one can search for 4 3-order cavities by 0-1 programming.

The main results of the new method (with all details included in Table S2) are as follows:

1. All-order spanning trees in the network are obtained, where the number of simplices in the *k*-order spanning tree is *r_k_*, *k* =1, 2, *•••*, *7*.

2. Morse function values and critical simplices in the network are assigned, where the numbers of the critical simplices in the *k*-simplices are *β_k_*, *k* = 0, 1, 2, 3, and the rest are zero.

3. Nested simplicial subnetworks ∅ ⊆ *K*_0_ ⊆ *K*_1_ ⊆ *•••* ⊆ *K*_4521_ = ***K*** in the network are obtained, where *n* = *r*_1_ + *β*_1_ + *r*_2_ + *β*_2_ + *r*_3_ + *β*_3_ + *r*_4_ + *•••* + *r*_7_ = 4,521.

4. Simplices composed of all-order cavities in the network and length distributions of cavities are obtained.

For example, the length distribution of 1-order cavities is shown in columns 2 to 8 of Table 1, while the optimal length distribution of 1-order cavities is shown in columns 9 and 10.

**Table 1.** Length distribution of 1-order cavities.

Length	4	5	6	7	8	9	10	4	5
Number	16	48	29	23	18	4	1	138	1

The optimal length distribution of 1-order cavities is obtained by the exhaustive searching method. First, according to the critical simplices, search all 1-order cycles with a length of 4 that pass through each given critical simplex. Then, select those cycles that are linearly independent as cavities and delete the corresponding critical simplices. Finally, increase the lengths of the searched cycles in the remaining critical simplices and search again, until all cavities are found.

The length distribution of 2-order cavities appears to be more complicated. Tracing the evolution of cavities from large to small will find the length distribution of 2-order cavities, as shown in Table 2. The total length of 121 2-order cavities is reduced from 3,330 to 1,790 2-order simplices.

**Table 2.** Length distribution of 2-order cavities.

Length	8	10	12	14	16	18	20	22	24	26	28	30	32	34	36	38	1,790
Number	27	28	15	12	3	8	6	6	2	1	1	5	1	1	3	2	121

The above is the result of 4 iterations. Each iteration is to add one 3-simplex or 2-cavity (with more than half of the same nodes) to it. Here, a 2-order cavity with length 34 is used as an example. After 2 iterations, the length of the cavity is reduced to 8, which achieves the best.

The 2-order cavity has nodes (2, 3, 4, 13, 14, 15, 17, 85, 87, 103, 114, 115, 117, 118, 121, 133, 143, 192); another 2-order cavity with length 30 has nodes (2, 3, 4, 13, 14, 15, 17, 85, 87, 103, 114, 115, 117, 121, 133, 143, 192), which share 27 same 2-simplices with the former, and they are deleted when 2 2-order cavities are added as shown by the crossed numbers shown in Table 3. Thus, the result yields a new 2-order cavity with length 10, which replaces the former. The new 2-order cavity has nodes (4, 13, 87, 117, 118, 133, 192); another 3-simplex has nodes (4, 13, 87, 118) to be added again. The result is a 2-order cavity with length 8, as shown in Table 3.

**Table 3.** Two iterations of a 2-order cavity with length 34.

No.	0-iteration	**+** 2-order cavity	1-iteration	**+** 3-simplex	2-iteration
1	117,118,192	117,133,192	117,118,192	4,13,87	117,118,192
2	4,13,118	4,13,87	4,13,118	4,13,118	13,87,133
3	4,87,118	13,15,133	4,87,118	4,87,118	13,117,118
4	13,15,133	13,117,133	13,87,133	13,87,118	87,118,192
5	13,87,133		13,117,118		87,133,192
6	13,117,118		87,118,192		117,133,192
7	87,118,192		87,133,192		13,117,133
8	87,133,192		117,133,192		13,87,118
9			4,13,87		
10			13,117,133		
*•••*					
35-36	4,13,87	This is a repeating simplex by Eq. 2 in the Discussion section.			

The length distribution of 3-order cavities is shown in columns 2 to 5 of Table 4, while the shorter length is shown in columns 6 and 7. The result is obtained by 0-1 programming [24], or through 5 iterations.

**Table 4.** Length distribution of 3-order cavities.

Length	16	20	24	31	16	28
Number	1	1	1	1	3	1

As can be seen above, the new method can quickly find simplices composed of all-order cavities in a sequence of nested subnetworks, but the length of the cavity is not necessarily the shortest. It needs to be shortened by multiple iterations.

### BA scale-free network [30]

**Fig. 4.**
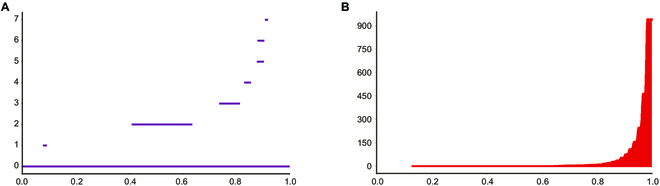
(A) Betti persistence barcodes of 0-order cavities. (B) Betti persistence barcodes of 1-order cavities [30].

In Ref. [30], initially there are 2 nodes in the network. Then, one new node with 2 new edges are added each time according to the BA model generation mechanism. When there are 1,000 nodes in the network, the process stops. Thus, a simplicial network ***K*** = {*V*, *E, T*} is obtained, in which the Euler characteristic number (and the Betti numbers *β_k_*) *χ* **=** 1,000 – 1,996 + 55 (**=** 1 − 942) **=** −941. The numbers of critical simplices obtained by Kannan's assignment method [30] are *c*_0_ = 8, *c*_1_ = 949, respectively, and the Betti persistence barcodes are obtained as shown in Fig. 4.

The abscissa axis in Fig. 4 is the normalized filtration weight dividing with *w_N_*, where *w_N_* is 1 plus the maximum value among weights assigned to the simplices in *K*.

In the simulation of Ref. [30], 55 2-order simplices are obtained, while our simulation result is 59 due to randomness, in which the Euler characteristic number (and the Betti numbers *β_k_*), *χ* **=** 1,000 – 1,996 + 59 (**=** 1 − 938) **=** −937, and the ranks of boundary matrices ***B***_1_, ***B***_2_ for describing the relationship between simplices are *r*_1_ = 999 and *r*_2_ = 59, respectively. The sequences of nested simplicial subnetworks, ∅ ⊆ *K*_0_ ⊆ *K*_1_ ⊆ *•••* ⊆ *K_n_* = ***K***, of this BA scale-free network ***K*** are *n*_1_ (**=** *c*_0_ + *c*_1_ − 1 = 17 + 954 − 1) = 970 and *n*_2_ (**=** *r*_1_ + *β_k_* + *r*_2_ **=** 999 + 938 + 59) = 1,996, obtained by Kannan's [30] and by our assignment method, respectively. The numbers of critical simplices obtained by 2 assignment methods are *c*_0_ = 17, *c*_1_ = 954 and *c*_0_ = 1, *c*_1_ = 938, respectively. Obviously, our assignment method can reach the minimum value and find the representative cycles of cavities (all details are given in Table S3).

### Stanford dragon graphic network [34]

**Fig. 5.**
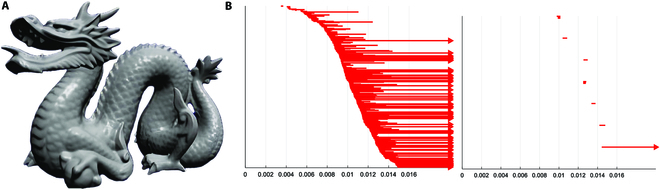
(A) Reconstruction of the Stanford dragon. (B) Betti persistence barcodes of 1- and 2-order cavities [34].

Point-cloud data of the network sample points are obtained uniformly at random from the 3-dimensional scans of the dragon photo, with reconstruction as shown in Fig. 5A. The point-cloud data list 1,000 points in the (*x*, *y*, *z*)-coordinates. As the threshold increases from 0 to 0.016, the threshold values of all simplices in the previous ordering are recorded. The simplicial network ***K*** finally formed by the point-cloud data has 1,000 0-simplices (nodes), 6,971 1-simplices (edges), 22,712 2-simplices (triangles), and 51,543 3-simplices (tetrahedrons). Its Euler characteristic number (and Betti numbers *β_k_*) is *χ* **=** 1,000 – 6,971 + 22,712 – 51,543 (**=** 1 – 66 + 1 – 34,738) **=** −34,802. The numbers of critical simplices obtained by Otter's assignment method [34] are *c*_0_ = 1,000, *c*_1_ = 276 + 999 = 1,275, *c*_2_ = 7 + 210 = 217, *•••*, and Betti persistence barcodes of 1- and 2-order cavities calculated by the method in Ref. [34] are shown in Fig. 5B. The lines with arrow in the picture are persistent, corresponding to 1- and 2-order cavities, and the rest represent birth and death.

Our method can also find all the above results with noise removed; i.e., there are no barcodes of birth and death therein. The reason is that the nested sequences of the 2 (space and simplicial) filtrations are different. Also, the representative cycles of the only 2-order cavity have lengths 38 [34] and 14 (our method), respectively. All details are given in Table S4.

In the above 3 case studies, the first example demonstrates that using spanning trees of various orders, it is possible to more easily explore higher-order network topologies from low to high; the second example shows that the network density can remain balanced as the network evolves and grows to get a filtration; the third example illustrates that increasing the threshold values will change the network from spare to dense, thereby becoming subtle and more accurate. All these examples from data science have nested simplicial subnetworks. It can be seen that, in order to study the topological characteristics of these datasets and their networks, critical simplices and Betti numbers that describe cavities are important and fundamental. Compared with the existing methods, our method can find the minimum number of critical simplices, and can also determine the number of nested simplicial subnetworks. In particular, our method can quickly find the simplices composed of all-order cavities, facilitating the understanding of the topological features of the dataset under investigation.

To further illustrate the significance of our method, consider the construction of a brain network model. Many research reports have shown that constructing the brain network model based on a fixed threshold of connectivity led to controversial results with unconvincing comparisons due to the lack of a priori biological knowledge and universally acceptable criteria. Our method does not pre-set any threshold value, but is dynamically increasing the threshold to reveal the topological characteristics of the network. This helps observe the evolution of the brain topology to obtain the best possible threshold value for the model, which is clearly related to the network order. This also helps reveal the topological characteristics of persistent homology, which is related to the number of critical simplices, thereby finding the essential differences of various brain network models. Moreover, the threshold defined in our method can be the distance between nodes, the coupling strength, or the gray level of data, indicating its usefulness in a broad range of real-world applications.

## Discussion

A filtered network is a nested sequence of its subnetworks: ∅ ⊆ *K*_0_ ⊆ *K*_1_ ⊆ *•••* ⊆ *K*_***n***_ = ***K***. The filtered network is the core in topological data analysis. Therein, a filtered simplicial network induced by Morse function is particularly important. Because the persistence barcodes calculated from different nested sequences are generally different, the assigned Morse function value (or the filtration obtained by other methods) determines the number of critical simplices, the birth and death time of some cavities, and the evolution of the cavity length (from large to small).

A *k*-cavity in the homology group *Z_k_* /*Y_k_* is a cycle in one of the linearly independent cycle-equivalent classes. Each class selects a cycle as its representative, and all representative cycles constitute a homology basis. A cavity is called optimal if the length of its representative cycle is the smallest, and a homology basis is called optimal if the total length of its representative cycles is the smallest. All the cavities obtained by other methods for the *C. elegans* neural network are not optimal. The optimal 1-order cavities are obtained by exhaustive searching, but the optimal 3-order cavities are obtained by the 0-1 programming method. For *k* > 1, the problem of computing an optimal homology basis is NP-hard [35]. Nevertheless, the problem is polynomial-time solvable for *k* = 1 [36]. For an *l*-order simplicial network, without counting for the iteration of simplifying the cavity lengths, the computational complexity of the proposed method of solving equations is *O*(*N*^3^), where *N* is the network size, i.e., *N* **=** *m*_0_ *+ m*_1_ + *m*_2_ *+ m*_3_ *+* ⋯+ *m_l_*, the total number of all-order simplices.

In the matrix equation (Eq. 4), because the matrix (*T − **B**_k_*)*^T^*(*T − **B**_k_*) is not reversible, another method is used to solve it. To do so, consider a *k*-order orientated simplex [*i*_0_, *i*_1_, ⋯ , *i_n_*] with a boundary [*i*_0_, *i*_1_, ⋯ , *i*_*p*-1_, *i*_*p*+1_, ⋯ , *i_n_*] where *i_p_* is removed. Then, each element of *B_n_* is given a plus or a minus sign [19], determined by (***−***1)*^p^*, and the resultant matrix is denoted as *B*_[*n*]_. All *k*-order cavities satisfy the following matrix equation:

$$\left(T-B_{\left[k\right]}\right){\left(T-x\right)}^{T}=\left(C-B_{\left[k\right]}\right)$$ (1)

Further, because the matrix (*T − **B***_[*k*]_)*^T^*(*T − **B***_[*k*]_) is invertible, the matrix equation (Eq. 1) has a unique solution, given by

$${\left(T-x\right)}^{T}{\left[{\left(T-B_{\left[k\right]}\right)}^{T}\left(T-B_{\left[k\right]}\right)\right]}^{-1}{\left(T-B_{\left[k\right]}\right)}^{T}\left(C-B_{\left[k\right]}\right)$$ (2)

Because of different definitions of chain and cycle [19], the method would cause the problem of repeating simplices in some cycles; see the last line in Table 3. It is more appropriate to use the following formula:

$$\begin{array}{cc} {\left(T-x\right)}^{T}= & {\left[{\left(T-B_{\left[k\right]}\right)}^{T}T-B_{\left[k\right]}\right]}^{-1}\\ & {\left(T-B_{\left[k\right]}\right)}^{T}\left(C-B_{\left[k\right]}\right)\left(\mathrm{mod}\cdot 2\right) \end{array}$$ (3)

As mentioned in Ref. [34], changing the coefficient field in the definitions of chain and cycle can affect the Betti numbers. For example, if one computes the homology of the Klein bottle with coefficients in the field ***F****_p_*, where *p* is a primer, then *β*_0_(*K*) = 1 for all primers *p*. However, *β*_1_(*K*) = 2 and *β*_2_(*K*) = 1 if *p* = 2, but *β*_1_(*K*) = 1 and *β*_2_(*K*) = 0 for all other primers *p*.

With the orientated boundary matrix, the Hodge-Laplacian matrix can be obtained by $L_{\left(n\right)}=B_{\left[n\right]}^{T}B_{\left[n\right]}+B_{\left[n+1\right]}B_{\left[n+1\right]}^{T}=L_{\left(n\right)}^{\text{down}}+L_{\left(n\right)}^{\mathrm{up}}$. It is known that the number of zero eigenvalues of the Hodge-Laplacian matrix *L*_(*k*)_ equals the number of *k*-order cavities, namely, the Betti number *β_k_*. Note that zero eigenvectors can provide information about cavities, which is a topic worth studying.

## Methods

Although homology calculation involves classifying cycles of a simplicial network, one can change the perspective to consider spanning trees of the network instead. This is because each new simplex added to a spanning tree will create a unique cycle. To ensure that the generated cycle is a cavity rather than just another simplex of higher order, the newly added simplex must be a critical one. For this purpose, an optimal method of assigning Morse values not only has to keep the persistence of cavities but also needs to reduce their calculations. It will be shown that the new method for solving the system of spanning trees and critical simplex equations can greatly improve the computational efficiency, e.g., compared to the 0-1 programming method used for searching all cycles [24].

### Basic concepts

A *k*-simplex *α_k_* is a fully connected subnetwork composed of *k* + 1 nodes, denoted by (*v*_0_, *v*_1_, *•••* , *v_k_*). Let *p* and *q* be 2 integers. A simplex *α_p_* is a face of another simplex *α_q_* if *α_p_* ⊂ *α_q_*. A simplex *α_q_* is a coface of another simplex *α_p_* if *α_q_* ⊃ *α_p_*. A simplicial complex ***K*** satisfies that (a) its every node is in some simplex in ***K***, and (b) for a simplex *α* ∈ ***K***, if a simplex *β* ⊂ *α*, then *β* ∈ ***K***. A conventional network is denoted by *G* **=** {*V*, *E*}, where *V* is the node set and *E* is the edge set, which has an adjacency matrix to characterize the connected edges. When all-order simplices of a network are enumerated or calculated, the network is called a simplicial network and denoted as ***K*** = {*V*, *E*, *T*, *•••*}, which has many incidence matrices, for example, boundary matrices ***B***_1_, ***B***_2_, etc., to describe the relationship between simplices [20]. A filtration of a simplicial network is defined as a sequence of nested simplicial subnetworks, ∅ ⊆ *K*_0_ ⊆ *K*_1_ ⊆ *•••* ⊆ *K_n_* = ***K***.

Let *C_k_* be the vector space in the binary field with a basis consisting of *k*-simplices, whose dimension *m_k_* is equal to the number of *k*-simplices. In the binary field, the addition between 2 vectors *c* and *d* is defined by set operations as *c* + *d* **=** (*c*∪*d*) − (*c*∩*d*). To study the linear dependence, boundary matrices are introduced. For instance, in vector space *C*_1_, define a node-edge matrix *B*_1_, in which an entry is 1 if a node is in the corresponding edge; otherwise, it is 0. The rank *r_k_* (*r*_0_ **=** 0 by convention) of the boundary matrix *B_k_* is the number of linearly independent vectors in space *C_k_*. Furthermore, define a boundary operator ∂*_k_*: *C_k_*→*C*_*k*-1_ to connect 2 successive spaces. Linear combinations of elements in *C_k_* are called *k*-chains. A *k*-cycle *l* is defined by ∂*_k_*(*l*) **=** 0. Moreover, define the kernel space of *C_k_* by ker(∂*_k_*) **=** {*l∈ C_k_* | ∂*_k_*(*l*) **=** 0}, and denote it as *Z_k_*. Also, define the image space on *C_k_* by im(∂_*k*+1_) **=** ∂_*k*+1_(*l*) | *l∈C*_*k*+1_}, which is the image of *C*_*k*+1_ mapping to *C_k_* and denote it as *Y_k_*. Since ∂*_k_*(∂_*k*+1_) **=** 0, one has im(∂_*k*+1_) ⊆ ker(∂*_k_*). Two *k*-cycles *c* and *d* are said to be equivalent, denoted *c* ~ *d*, if *c* + *d* is a boundary of a (*k* + 1)-chain. Classifying the kernel (cycle) space *Z_k_* with respect to *Y_k_* yields a homology group *Z_k_*/*Y_k_*. A *k*-cavity is a cycle that is usually selected with the shortest length in one of the linearly independent cycle-equivalent classes, whose number is equal to the Betti number *β_k_* **=** *m_k_* − *r_k_* − *r*_*k*+1_.

A simplicial network containing no *k*-cycle is called a forest, while a connected forest is called a *k*-tree. If the network itself is not a *k*-tree, but it can be seen as a certain *k*-tree with some additional simplices of older not higher than *k*, then this *k*-tree is called a *k*-order spanning tree. In this paper, the *k*-order spanning tree is searched by finding the rank of the boundary matrix ***B****_k_*, for which details are given in Table S1.

Now, let ***K*** be a simplicial network. Given a function *f***: *K***→*R*, for each simplex *α_p_* ∈ ***K***, define 2 sets of simplices: *U^f^*(*α_p_*) **=** {*α*_*p+*1_**|***α*_*p+*1_ ⊃ *α_p_*, *f*(*α*_*p+*1_) ≤ *f*(*α_p_*)} and *V ^f^*(*α_p_*) **=** {**|***α*_*p-*1_ ⊂ *α_p_*, *f*(*α*_*p-*1_) ≥ *f*(*α_p_*)}. Then, a function *f***: *K***→*R* is a discrete Morse function if and only if for every simplex *α_p_* ∈ ***K***, both #*U^f^*(*α_p_*) ≤ 1 and #*V ^f^*(*α_p_*) ≤ 1 hold. The symbol # indicates the number of elements in the set. That is, at most one coface of one order higher is allowed such that *f*(*α*_*p+*1_) ≤ *f*(*α_p_*) and at most one face of one order lower is allowed such that *f*(*α*_*p-*1_) ≥ *f*(*α_p_*). A simplex *α_p_* of a simplicial network ***K*** with a discrete Morse function *f* is critical if and only if both #*U^f^*(*α_p_*) **=** 0 and #*V ^f^*(*α_p_*) **=** 0 hold. Let *c_k_* be the number of *k*-order critical simplices. Then, for an *l*-order simplicial network, one has *m_k_* ≥ *c_k_* ≥ *β_k_* when 0 ≤ *k* ≤ *l* and ***χ* =** *m*_0_ *− m*_1_ + *m*_2_ *− m*_3_ *+*⋯+ (*−*1)*^l^m_l_* **=** *c*_0_ *− c*_1_ *+ c*_2_ *− c*_3_*+*⋯+(*−*1)*^l^c_l_* **=** *β*_0_ *− β*_1_ *+ β*_2_ *− β*_3_ *+*⋯+(*−*1)*^l^β_l_*.

If the function defined on a simplicial network ***K*** can induce a sequence of nested simplicial subnetworks, ∅ ⊆ *K*_0_ ⊆ *K*_1_ ⊆ *•••* ⊆ *K_n_* **= *K***, then this sequence is called a filtered simplicial network. A *k*-cavity that appears in the subnetwork *K_i_* can potentially become the boundary of a (*k* + 1)-chain of a later subnetwork *K_i+j_* with *j* > 0, which will no longer constitute a *k*-cavity in *K_i+j_*. As such *k*-cavity has a unique index that corresponds to its birth (*i* of *K_i_*) and death (*i+j* of *K_i+j_*) in crossing the filtration, the other *k*-cavities without dead indexes are said to be persistent.

Now, consider the network with 7 0-simplices (nodes), 10 1-simplices (edges), and 3 2-simplices (shaded triangles), as shown in Fig. 3A. The characteristic number of this 2-order simplicial network ***K*** is *χ =* 7 *–* 10 + 3 = 0. Next, specify the Morse values to the simplex for the network in the following way [32]: 1-*v*_0_, 2-*v*_1_, (1,2)-*e*_1_, 3-*v*_2_, 4-*v*_3_, (3,4)-*e*_3_, 5-*v*_4_, (4,5)-*e*_4_, 6-*v*_5_, (5,6)-*e*_5_, (1,2)-*e*_6_, (4,6)-*e*_7_, (4,5,6)-*f*_7_, 7-*v*_8_, (3,7)-*e*_8_, (4,7)-*e*_9_, (3,6)-*e*_10_, (2,6)-*e*_11_, (2,3,6)-*f*_11_, and (3,4,7)-*f*_12_. Thus, the network has 2 nodes *v*_0_ and *v*_2_; 3 edges *e*_6_, *e*_9_, and *e*_10_; and 1 face *f*_12_, which are critical simplices. The summation of the numbers of critical simplices with alternative signs, 2 *–* 3 + 1 =0, is also equal to the characteristic number *χ*. The sequence of nested simplicial subnetworks of this network ***K*** is ∅ ⊆ *K*_0_ ⊆ *K*_1_ ⊆ *•••* ⊆ *K*_12_ = ***K***. For example, *K*_2_ = {*v*_0_, *v*_1_, *e*_1_, *v*_2_}. Non-diamond arrow lines in Fig. 3B show the corresponding Betti persistence barcodes, where *v*_0_ and *e*_10_ are persistent, while *v*_2_ and *e*_9_ are birth and death elements. That is, *v*_0_ and *e*_10_ are cavities of order 0 and 1, respectively. The starting time is indicated by the subscript. Here, *v*_2_ shows that the birth time is 2, *e*_6_ shows that the death time is 6, *e*_9_ shows that the birth time is 9, and *f*_12_ shows that the death time is 12.

However, different methods of assigning values may result in different starting time and also different sizes of a cavity. The red diamond arrow lines in Fig. 3B show the Betti persistence barcodes of the corresponding Morse values: 1-*v*_0_, 2-*v*_1_, (1,2)-*e*_1_, 3-*v*_2_, (2,3)-*e*_2_, 4-*v*_3_, (3,4)-*e*_3_, 5-*v*_4_, (4,5)-*e*_4_, 6-*v*_5_, (5,6)-*e*_5_, 7-*v*_6_, (3,7)-*e*_6_, (3,6)-*e*_7_, (4,6)-*e*_8_, (4,5,6)-*f*_8_, (4,7)-*e*_9_, (3,4,7)-*f*_9_, (2,6)-*e*_10_, and (2,3,6)-*f*_10_. Clearly, there are only 2 critical simplices: node *v*_0_ and edge *e*_7_. Thus, the figure between critical simplices and their subscript numbers is just a persistence barcode. The summation of the numbers of critical simplices with alternative signs, 1 − 1 = 0, is also equal to the characteristic number *χ*. The sequence of nested simplicial subnetworks is ∅ ⊆ *K*_0_ ⊆ *K*_1_ ⊆ *•••* **⊆** *K*_10_ = ***K***.

The above new Morse values show that the original *e*_10_ comes before *e*_7_, the 1-order cavity starting with 4 edges, but after joining by *e*_8_, it has only 3 edges. This means that the 1-order cavity appears earlier and then shrinks from big to small.

### An example

**Fig. 6.**
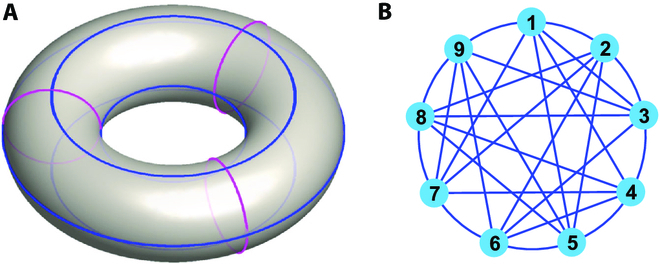
(A) A torus example. (B) The obtained network with 9 nodes and 27 edges.

A torus with small red cycles and big blue cycles is shown in Fig. 6A for illustration. Consider a network of 3 small red cycles and 3 big blue cycles, which has 9 nodes and 18 edges.

A triangulation of the torus can be obtained by adding 9 edges to the quadrilateral in the network. The obtained network with 9 nodes and 27 edges is shown in Fig. 6B. The simplicial network composed of all-order simplices in the network is obtained as ***K* =** {9 0-simplices (nodes), 27 1-simplices (edges), 18 2-simplices (triangles)}. Its characteristic number *χ* = *m*_0_ – *m*_1_ + *m*_2_ = 9 − 27 + 18 = 0. The boundary matrices ***B***_1_, ***B***_2_, etc. describe the relationship between simplices. The 18 2-order simplices of ***K*** are as follows: (1,2,5), (1,2,7), (1,3,4), (1,3,9), (1,4,5), (1,7,9), (2,3,6), (2,3,8), (2,5,6), (2,7,8), (3,4,6), (3,8,9), (4,5,8), (4,6,7), (4,7,8), (5,6,9), (5,8,9), and (6,7,9). However, triangles (1,2,3), (1,4,7), (1,5,9), (2,5,8), (2,6,7), (3,4,8), (3,6,9), (4,5,6), and (7,8,9) are not 2-simplices in ***K***.

1. Searching all-order spanning trees in a network

There are many ways to find spanning trees in a given network. Here, the row elementary transformation method of matrix is used to find the rank of the boundary matrices ***B***_1_ and ***B***_2_. Start from the last row of the boundary matrix ***B***_1_ to find the leftmost 1, and go up row by row. If 2 different rows in the same column are both 1, by the row transformation over binary operations, they will be removed. The desired spanning tree is finally obtained, as can be seen from the columns corresponding to all “1” in boldface (may be an identity matrix) shown in Table 5.

**Table 5.** Solving matrix (Eq. 4) by row operations over the binary field.

	*T **−*** ***B***_**1**_								(*T **−*** ***x***)*^T^*	*C − **B***_**1**_	
	(1,2)	(1,3)	(1,4)	(1,5)	(1,7)	(1,9)	(2,6)	(2,8)		(2,3)	(4,7)
1	1	1	1	1	1	1	0	0		0	0
2	**1**	0	0	0	0	0	1	1	*x*_(1,2)_	1	0
3	0	**1**	0	0	0	0	0	0	*x*_(1,3)_	1	0
4	0	0	**1**	0	0	0	0	0	*x*_(1,4)_	0	1
5	0	0	0	**1**	0	0	0	0	*x*_(1,5)_	0	0
6	0	0	0	0	0	0	**1**	0	*x*_(1,7)_	0	1
7	0	0	0	0	**1**	0	0	0	*x*_(1,9)_	0	0
8	0	0	0	0	0	0	0	**1**	*x*_(2,6)_	0	0
9	0	0	0	0	0	**1**	0	0	*x*_(2,8)_	0	0

The 1-order spanning tree of the triangulation network consists of (1,2), (1,3), (1,4), (1,5), (1,7), (1,9), (2,6), and (2,8). This shows that *r*_1_ = 8.

The 2-order spanning tree of the triangulation network consists of (1,2,5), (1,2,7), (1,3,4), (1,3,9), (1,4,5), (1,7,9), (2,3,6), (2,3,8), (2,5,6), (2,7,8), (3,4,6), (3,8,9), (4,5,8), (4,6,7), (4,7,8), (5,6,9), and (5,8,9). This shows that *r*_2_ = 17. Thus, *β*_0_ = 9 – 0 – 8 = 1, *β*_1_ = 27 – 8 – 17 = 2, and *β*_2_ = 18 – 17 – 0 = 1.

2. Classifying the remaining simplices other than spanning trees

Except for the simplices in the 1-order spanning tree, the remaining 1-simplices can be classified into 2 categories: simplices in the 2-order spanning tree and 1-order cavity-generating simplices. For example, 1-simplices (2,3) and (4,7) are not in the 2-order spanning tree. Thus, they are 1-order cavity-generating simplices. Similarly, the 2-simplex (6,7,9) is a 2-order cavity-generating simplex. Obviously, the first selected node 1 is a 0-order cavity-generating simplex.

3. Assigning Morse function values to simplices

To find the lower bound of the critical simplex numbers, it should be noted that only those cavity-generating simplices can be critical simplices. Therefore, the function values are specified to the simplices in the following way: 1-*v*_0_, 2-*v*_1_, (1,2)-*e*_1_, 3-*v*_2_, (1,3)-*e*_2_, 4-*v*_3_, (1,4)-*e*_3_, 5-*v*_4_, (1,5)-*e*_4_, 7-*v*_5_, (1,7)-*e*_5_, 9-*v*_6_, (1,9)-*e*_6_, 6-*v*_7_, (2,6)-*e*_7_, 8-*v*_8_, (2,8)-*e*_8_, (2,3)-*e*_9_, (4,7)-*e*_10_, (2,5)-*e*_11_, (1,2,5)-*f*_11_, (2,7)-*e*_12_, (1,2,7)-*f*_12_, (3,4)-*e*_13_, (1,3,4)-*f*_13_, (3,9)-*e*_14_, (1,3,9)-*f*_14_, (4,5)-*e*_15_, (1,4,5)-*f*_15_, (7,9)-*e*_16_, (1,7,9)-*f*_16_, (3,6)-*e*_17_, (2,3,6)-*f*_17_, (3,8)-*e*_18_, (2,3,8)-*f*_18_, (5,6)-*e*_19_, (2,5,6)-*f*_19_, (7,8)-*e*_20_, (2,7,8)-*f*_20_, (4,6)-*e*_21_, (3,4,6)-*f*_21_, (8,9)-*e*_22_, (3,8,9)-*f*_22_, (5,8)-*e*_23_, (4,5,8)-*f*_23_, (6,7)-*e*_24_, (4,6,7)-*f*_24_, (4,8)-*e*_25_, (4,7,8)-*f*_25_, (6,9)-*e*_26_, (5,6,9)-*f*_26_, (5,9)-*e*_27_, (5,8,9)-*f*_27_, and (6,7,9)-*f*_28_.

The subscripts of critical simplices, 1-*v*_0_, (2,3)-*e*_9_, (4,7)-*e*_10_, and (6,7,9)-*f*_28_, indicate the starting time of 1 0-order cavity, 2 1-order cavities, and 1 2-order cavity, respectively.

This Morse function induces a sequence of nested simplicial subnetworks, ∅ ⊆ *K*_0_ ⊆ *K*_1_ ⊆ *•••* ⊆ *K*_28_ = ***K***, for the triangulation network. It will greatly simplify the homology calculation for lower orders.

4. Computing simplices composed of all-order cavities

A *k*-cavity can be expressed as ***x* =** (*x*_1_, *x*_2_, *•*, *x_m_k__*) ∈ *C_k_*, in which each component *x_i_* takes value 1 or 0, where 1 represents a *k*-simplex with index *i* in the cavity while 0 means no such simplex exists. Let ***B****_k_* be the boundary matrix between the (*k* − 1)-simplices and the *k*-simplices. Then, a *k*-cavity ***x*** must satisfy the equation ***B****_k_****x****^T^* **=** 0 (mod 2).

The *j*th column of matrix ***B****_k_* is marked as ***B****_k_^j^*, and the columns in the matrix obtained by removing the *j*th column from ***B****_k_* is denoted as ***B****_k_*^-*j*^. Suppose that the *j*th simplex is a *k*-order cavity-generating simplex. From the equation ***B****_k_****x****^T^* **=** 0 (mod 2), with multiplication according to block matrices, it follows that ***B****_k_*^−*j*^(***x***^−*j*^)*^T^* = −***B****_k_^j^* (mod 2). The matrix composed of all columns of the *k*-order spanning tree and the matrix composed of all columns of the corresponding *k*-order cavity-generating simplices in matrix ***B****_k_* are denoted as *T − **B**_k_* and *C − **B**_k_*, respectively. Let *T − **x*** express the unknown 0-1 matrix of simplices corresponding to the *k*-order spanning tree. Then, all simplices of the *k*-order cavities satisfy the following matrix equation:

$$\left(T-Bk\right){\left(T-x\right)}^{T}=\left(C-Bk\right)\left(\mathrm{mod}\cdot 2\right)$$ (4)

Note, however, that although the spanning tree matrix (*T − **B**_k_*) is of full rank, the inverse of the matrix (*T − **B**_k_*)*^T^*(*T − **B**_k_*) may not exist because of the relationship between matrix operations over the binary field; thus, one can only use the row operation of the matrix elementary transformation to solve this matrix equation.

From Table 5, one obtains the solution of matrix equation (Eq. 4): *x*_(1,2)_ = (1.0), *x*_(1,3)_ = (1,0), *x*_(1,4)_ = (0,1), *x*_(1,5)_ = (0,0), *x*_(1,7)_ = (0,1), *x*_(1,9)_ = (0.0), *x*_(2,6)_ = (0.0), *x*_(2,8)_ = (0.0). Thus, 2 1-order cavities are obtained: {(1,2), (1,3), (2,3)} and {(1,4), (1,7), (4,7)}, respectively. Similarly, a 2-order cavity consists of {(1,2,5), (1,2,7), (1,3,4), (1,3,9), (1,4,5), (1,7,9), (2,3,6), (2,3,8), (2,5,6), (2,7,8), (3,4,6), (3,8,9), (4,5,8), (4,6,7), (4,7,8), (5,6,9), (5,8,9), (6,7,9)}. This 2-order cavity is obtained by similarly solving a system of equations, which also yields a triangulation of the torus.

All computations and tables of this example are presented in Table S1.

### Method summary

The method developed in this paper is an optimal method of assigning Morse values to a given simplicial network. The method starts from any node in a connected branch and assigns it a value of 0; searches all-order spanning trees and identifies critical simplices with the boundary matrix ***B****_k_*; finds the node and edge connected to the start node in 1-order spanning tree and assigns a value of PO to the node and edge until all edges in the spanning tree are exhausted, then assigns the value of 1-order critical simplex one by one until the last *l*-order simplex is reached for an *l*-order simplicial network; gets the nested simplicial subnetworks ∅ ⊆ *K*_0_ ⊆ *K*_1_ ⊆ *•••* ⊆ *K_n_* = ***K***, where *n* = *r*_1_ + *β*_1_ + *r*_2_ + *β*_2_ + *•••* + *•••* + *r_l_* + *β_l_*. Then, the method solves the equation systems of spanning trees and critical simplices from lower order to higher order, and finally obtains simplices of all *k*-order cavities by row operations on a matrix elementary transformation in the binary field.

## Data Availability

Data are available in the Supplementary Materials section.

## Supplementary Materials

Supplementary 1 Supplementary information is provided in the Appendix. Appendix: Supplementary Information (Tables S1 to S4) Table S1. Torus triangulation network. Table S2. *C. elegans* neural network [33]. Table S3. BA scale-free model network [30]. Table S4. Stanford dragon graphic network [34]. Data of Tables S1 to S4 are available at https://github.com/ChuangMa1900/Supplementary-Information-Tables-S1-to-S4.git

## References

[1] Morley-Fletcher, R. Big data: What is it and why is it important? In: *Digital agenda for Europe.* European Commission. TechTarget/Data Management, online; 2013.

[2] Carlsson G. Topology and data. Bull Am Math Soc. 2009;46:255–308.

[3] Offroy M, Duponchel L. Topological data analysis: A promising big data exploration tool in biology, analytical chemistry and physical chemistry. Anal Chim Acta. 2016;910:1–11. 26873463 10.1016/j.aca.2015.12.037

[4] Newman M. *Networks*. Oxford (UK): Oxford University Press; 2018.

[5] Edelsbrunner H, Harer J. *Computational topology: An introduction*. *Applied Mathematics*. Boston (MA): American Mathematical Society; 2010.

[6] Dey T, Wang Y. *Computational topology for data analysis*. Cambridge (UK): Cambridge University Press; 2022.

[7] Edelsbrunner H, Letscher D, Zomorodian A. Topological persistence and simplification. Discrete Comput Geom. 2002;28:511–533.

[8] Carlsson G, Zomorodian A. Computing persistent homology. Discrete Comput Geom. 2005;33:249–274.

[9] Ghrist R. Barcodes: The persistent topology of data. Bull Am Math Soc. 2008;45(1):61–75.

[10] Edelsbrunner H, Harer J. Persistent homology—A survey. Contemp Math. 2008;453:257–282.

[11] Hatcher A. *Algebraic topology*. Cambridge (UK): Cambridge University Press; 2002.

[12] Zomorodian A. Fast construction of the Vietoris-rips complex. Comput Graph. 2010;34:263–271.

[13] Edelsbruneer H, Kirkpatrick D, Seidel R. On the shape of a set of points in the plane. IEEE Trans Inform Theory. 1983;29(4):551–559.

[14] Chazal F, De Silva V, Oudot S. Persistence stability for geometric complexes. Geom Dedicata. 2014;173:193–214.

[15] Lehmberg D, Dietrich F, Köster G, Bungartz HJ. Datafold: Data-driven models for point clouds and time series on manifolds. J Open Source Softw. 2020;5(51):2283.

[16] Watts D, Strogatz S. Collective dynamics of 'small-world' networks. Nature. 1998;393:440–442. 9623998 10.1038/30918

[17] Barabási AL, Albert R. Emergence of scaling in random networks. Science. 1999;286(5439):509–512. 10521342 10.1126/science.286.5439.509

[18] Shi D, Lü L, Chen G. Totally homogeneous networks. Natl Sci Rev. 2019;6(5):962–969. 34691957 10.1093/nsr/nwz050 PMC8291615

[19] Bianconi, G. *Higher-order networks: An introduction to simplicial complexes*. Cambridge (UK): Cambridge University Press; 2020.

[20] Shi D, Chen G. Simplicial networks: A powerful tool for characterizing higher-order interactions. Natl Sci Rev. 2022;9(5): nwac038. 35547955 10.1093/nsr/nwac038 PMC9084355

[21] Battiston F, Cencetti G, Iacopini I, Latora V, Lucas M, Patania A, Young J-G, Petri G. Networks beyond pairwise interactions: Structure and dynamics. Phys Rep. 2020;874:1–92.

[22] Battiston F, Petri G. *Higher-order systems. Complexity*. Berlin (Germany): Springer; 2022.

[23] Fan T, Lü L, Shi D, Zhou T. Characterizing cycle structure in complex networks. Commun Phys. 2021;4(272):1–9.

[24] Shi D, Chen Z, Sun X, Chen Q, Ma C, Lou Y, Chen G. Computing cliques and cavities in networks. Commun Phys. 2021;4: 249.

[25] Morse M. *The calculus of variations in the large*. Boston (MA): American Mathematical Society; 1934.

[26] Barannikov S. Framed Morse complex and its invariants. Adv Sov Math. 1994;21:93–115.

[27] Forman R. Morse theory for cell complexes. Adv Math. 1998;134:90–145.

[28] Sizemore A, Giusti C, Bassett D. Classification of weighted networks through mesoscale homological features. J Complex Netw. 2016;5(2):245–273.

[29] Horak D, Maletić S, Rajković M. Persistent homology of complex networks. J Stat Mech Theory Exp. 2009;2009: P03034.

[30] Kannan H, Saucan E, Roy I, Samal A. Persistent homology of unweighted complex networks via discrete Morse theory. Sci Rep. 2019;9: 13817. 31554857 10.1038/s41598-019-50202-3 PMC6761140

[31] Mischaikow K, Nanda V. Morse theory for filtrations and efficient computation of persistent homology. Discrete Comput Geom. 2013;50(2):330–353.

[32] Scoville A. *Discrete Morse theory*. Boston (MA): American Mathematical Society; 2019.

[33] Rossi R, Ahned N. The network data repository with interactive graph analysis and visualization. In: *Twenty-Ninth AAAI Conference, Austin, TX, USA*. Palo Alto (CA): AAAI Press; 2015. p. 4292–4293.

[34] Otter N, Porter MA, Tillmann U, Grindrod P, Harrington HA. A roadmap f or the computation of persistent homology. EPJ Data Science. 2017;6(1):38. 10.1140/epjds/s13688-017-0109-5 PMC6979512 32025466

[35] Chen C, Freedman D. Hardness results for homology localization. Discrete Comput Geom. 2011;45(3):425–448.

[36] Dey TK, Sun J, Wang Y. Approximating loops in a shortest homology basis from point data. In: *Proceedings of the 26th Annual Symposium on Computational Geometry, Snowbird, UT, USA*. New York (NY): Association for Computing Machinery; 2010. p. 166–175.

