# Supplementary material for: Computing Persistent Homology by Spanning Trees and Critical Simplices
Source: Research (Wash D C). 2023 Sep 14;6:0230. doi: 10.34133/research.0230 (PMC10501364; doi:10.34133/research.0230)
Supplement: Supplementary 1 — Supplementary information is provided in the Appendix. Appendix: Supplementary Information (Tables S1 to S4) Table S1. Torus triangulation network. Table S2. C. elegans neural network [33]. Table S3. BA scale-free model network [30]. Table S4. Stanford dragon graphic network [34]. Data of Tables S1 to S4 are available at https://github.com/ChuangMa1900/Supplementary-Information-Tables-S1-to-S4.git [file research.0230.f1.docx]

**Supplementary information** is provided in the Appendix.

**Appendix: Supplementary Information (Tables S1 to S4)**

**Table S1: Torustriangulation network**

This table lists the Morse function values of all simplices, the spanning trees of the boundary matrixes ***B***_1_ and ***B***_2_, and the simplices composed of 1- and 2-order cavities for the network.

**Table S2: C. elegans neural network**^33^

This table lists the Morse function values of all simplices, the simplices composed of 1-, 2- and 3-order cavities, the iterative process of 2-order cavities, and an iterative example for the network.

**Table S3: BA scale-free model network**^30^

This table lists the Morse function values of all simplices, the simplices composed of 1-order cavities, and the results obtained by Kannan's^30^ method for the network which is simulated here.

Variables in Kannan's method are as follows:

DFM=Discrete Morse function values;

Flag=To keep track with the size of the set *U_α_*for each simplex *α*;

IsCritical=To indicate if a given simplex is critical;

FiltrationWeight=To store the filtration weight corresponding to each simplex.

**Table S4: Stanford dragon graphicnetwork**^34^

This table lists the points in the(*x*, *y*, *z*)-coordinates, the present thresholds of all simplices, the persistence barcodes of 1- and 2-order cavities calculated by **javaplex**in Ref. [34], the representative cycles with two lengths of the only 2-order cavity,and the Morse function values of all simplices obtained by the new method for the network.

Data of **Tables S1 to S4** are available:

<https://github.com/ChuangMa1900/Supplementary-Information-Tables-S1-to-S4.git>
